# A new pipeline for pathophysiological analysis of the mammary gland based on organoid transplantation and organ clearing

**DOI:** 10.1242/jcs.242495

**Published:** 2020-06-23

**Authors:** Emilie Lagoutte, Clémentine Villeneuve, Vincent Fraisier, Denis Krndija, Marie-Ange Deugnier, Philippe Chavrier, Carine Rossé

**Affiliations:** 1 Research center, Institut Curie, Paris Sciences et Lettres Research University, Sorbonne Université, CNRS (Centre National de la Recherche Scientifique), UMR 144, 26 rue d'Ulm, F-75005 Paris, France; 2 Institut national de la santé et de la recherche médicale, INSERM, Paris F-75013, France

**Keywords:** Mammary gland, Tissue clearing, Organoid transplantation, Cell dissemination, Atypical PKCι

## Abstract

Recent developments in techniques for tissue clearing and size reduction have enabled optical imaging of whole organs and the study of rare tumorigenic events *in vivo*. The adult mammary gland provides a unique model for investigating physiological or pathological processes such as morphogenesis or epithelial cell dissemination. Here, we establish a new pipeline to study rare cellular events occurring in the mammary gland, by combining orthotopic transplantation of mammary organoids with the uDISCO organ size reduction and clearing method. This strategy allows us to analyze the behavior of individually labeled cells in regenerated mammary gland. As a proof of concept, we analyzed the localization of rare epithelial cells overexpressing atypical protein kinase C iota (also known as PRKCI, referred to here as aPKCι) with an N-terminal eGFP fusion (GFP-aPKCι^+^) in the normal mammary gland. Using this analytical pipeline, we were able to visualize epithelial aPKCι^+^ cells escaping from the normal mammary epithelium and disseminating into the surrounding stroma. This technical resource should benefit mammary development and tumor progression studies.

## Abstract (precis)

**Summary:** A novel technical strategy combining orthotopic transplantation of mammary organoids, uDISCO organ size reduction and clearing, and fluorescence imaging enables visualization and investigation of rare cellular events *in vivo*.

## INTRODUCTION

The mammary epithelial bilayer is composed of luminal and myoepithelial cells, sitting on a basement membrane that separates the double epithelium from the surrounding stroma (Macias and Hinck, 2012). The mammary stroma, also known as the mammary fat pad, comprises multiple cellular elements, including adipocytes, fibroblasts, hematopoietic cells, lymphatic vessels and blood vessels.

Exploring individual cell behavior is crucial in the study of complex processes such as development or cancer metastasis as early cell dissemination, yet it remains challenging. Indeed, visualization of rare cellular events occurring in the context of the whole organ remains poorly compatible with classical histological techniques based on staining of thin tissue sections or even thicker tissue slices. Here, we describe a new pipeline exploiting the uDISCO method (Pan et al., 2016) for organ clearing and size reduction. We employed this technique to visualize rare events by using the remarkable regenerative properties of the mammary epithelium following orthotopic transplantation of epithelial fragments or organoids (Deome et al., 1959; Jarde et al., 2016). Combining these two powerful approaches enabled imaging of the whole mammary gland as a mosaic of confocal z-stacks of images, providing whole organ reconstruction with a cellular to subcellular resolution. This pipeline allowed us to analyze the global morphology of the gland and the composition of the microenvironment while detecting epithelial or stromal localization of fluorescently tagged cells previously incorporated into the transplanted mammary organoids.

## RESULTS

**Fig. 1.**
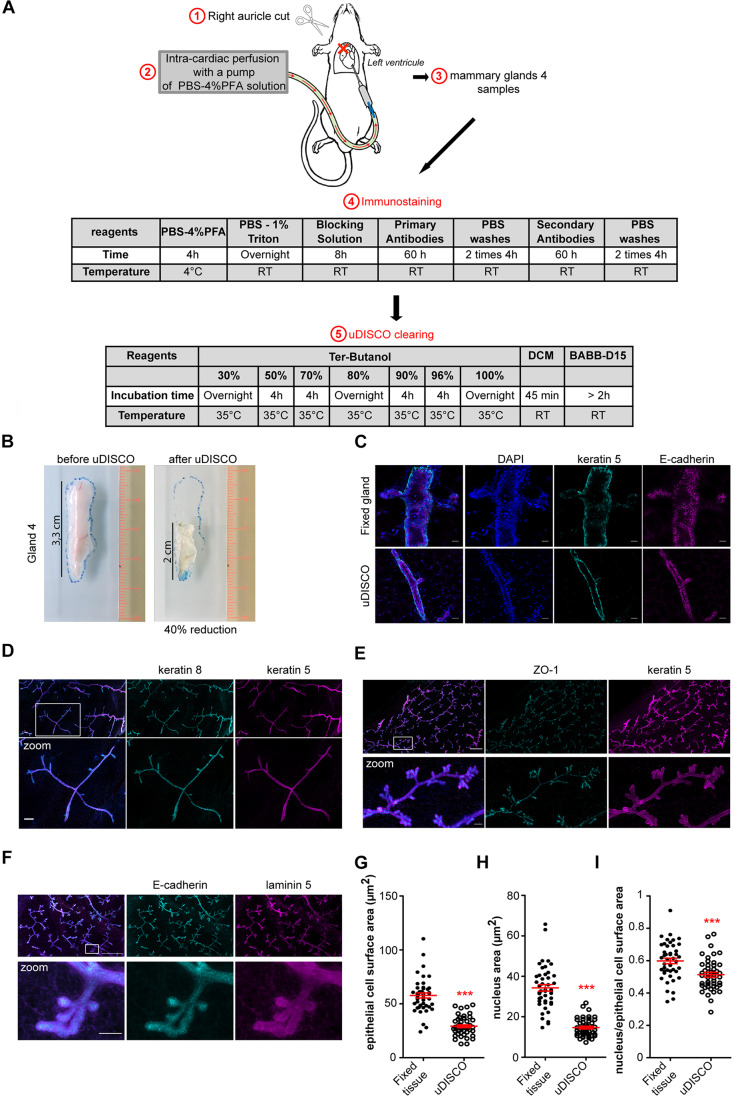
**Mouse mammary gland clearing using uDISCO technique.** (A) Experimental steps of the mouse mammary gland uDISCO clearing procedure. (B) Images of mammary gland #4 from a FVB wild-type mouse before and after uDISCO clearing. (C) Images of immunostained mouse mammary gland #4 after fixation (upper row) or fixation followed by uDISCO treatment (bottom row). Myoepithelial and luminal cells were stained with anti-keratin 5 (cyan) and anti-E-cadherin (magenta) antibodies, respectively. (D) Mosaic reconstruction of z-projection of 145 confocal planes with 2 µm interval (290 µm total thickness) of a cleared mouse gland #4. Myoepithelial and luminal epithelial cells were stained with anti-keratin 5 (magenta) and anti-keratin 8 (cyan) antibodies, respectively. (E) Mosaic reconstruction of z-projection of 102 confocal planes with 3 µm interval (306 µm total thickness) of a cleared mouse gland #4. Myoepithelial and luminal epithelial cells were stained with anti-keratin 5 (magenta) and anti-ZO1 (cyan) antibodies, respectively. (F) Mosaic reconstruction of z-projection of 90 confocal planes with 3 µm interval (270 µm total thickness) of cleared mouse gland #4. Luminal epithelial cells and the basement membrane were stained with anti-E-cadherin (cyan) and anti-laminin 5 (magenta) antibodies, respectively. (G,H) Quantification of epithelial cell surface area (G) or epithelial nucleus surface area (H) of fixed or fixed followed by uDISCO treatment of mouse mammary glands #4. The epithelial cell surface area and nucleus area were manually measured in the cell medium confocal plane, respectively, using E-cadherin and DAPI staining. (I) Ratio of epithelial nucleus surface area to epithelial cell surface area of fixed (fixed tissue) or fixed followed by uDISCO treatment (uDISCO) mouse mammary glands #4. For each condition, 45 cells from 3 independent experiments were quantified. Data are mean±s.e.m. Statistical significance was calculated using either a Mann–Whitney test (G,H) or an unpaired *t*-test (I); ***P<0.001. Scale bars: 20 µm (C), 100 µm (D), 500 µm (E,F upper row), 50 µm (E,F zoom).

We adapted the uDISCO technique (Pan et al., 2016) for analysis of the whole mammary gland. To improve the size reduction of the sample using clearing organic compounds that also preserved fluorescence and provided an efficient tissue-clearing effect, the uDISCO technique was preferred over previously described methods such as 3DISCO (Erturk et al., 2012; Chung et al., 2013) or iDISCO (Renier et al., 2014). For this purpose, an intracardiac perfusion was performed in the anesthetized animal to replace the blood of the animal with phosphate buffered saline (PBS) solution, followed by intracardiac fixation by injection of PBS containing 4% PFA (Fig. 1A, steps 1 and 2). Mouse #4 mammary glands were sampled (Fig. 1A, step 3), processed first for immunostaining (step 4) and then for size reduction and clearing (step 5). Clearing and a ∼40% reduction in size of the whole mammary gland were typically observed (Fig. 1B). We then analyzed whether the general organization and apico-basal polarity of the whole mammary epithelium were preserved by immunostaining (Fig. 1C-F; Movie 1). After reduction, we were able to image a large volume of the mammary gland by performing mosaic reconstruction of confocal z-stacks of images (Fig. 1D-F). The luminal epithelial cell layer (E-cadherin^+^ or keratin 8^+^) was surrounded by the myoepithelial cell layer (keratin 5^+^ or SMA^+^), outlined by the laminin 5-positive basement membrane (Fig. 1D,F; Fig. S1A, Movie 1). Thus, we concluded that organization of the double myoepithelial and luminal epithelial cell layers was preserved, as compared with classical immunostaining of tissue slices without uDISCO treatment (Fig. 1C,D; Movie 1). We could also detect apico-basal polarity markers of epithelial cells, such as ZO-1 and E-cadherin (Fig. 1E,F). The epithelial cell surface area and nucleus surface area were reduced by 50% (Fig. 1G,H). However, the ratio of nucleus to epithelial cell surface area was smaller after uDISCO treatment (ratio 0.51) than for non-cleared tissues (ratio 0.6), suggesting a slightly greater magnitude of nucleus versus cytoplasm size reduction (Fig. 1I). Together, our data show that uDISCO clearing of the mammary gland resulted in a twofold reduction in the size of the organ while preserving its organization. In addition, high organ transparency was achieved, enabling low-background immunostaining and deep-tissue imaging by classical confocal microscopy.

To extend our analysis to the detection of various proteins localized in the cleared mouse mammary gland, additional antibodies were tested (Fig. 1D,F; Fig. S1A). As previously established (Taddei et al., 2008; Macias and Hinck, 2012), we found smooth muscle actin (SMA) restricted to the basal myoepithelial cell layer and CD29 (β1-integrin) enriched in this layer, which was overall positive for keratin 14 (Fig. S1A,B). In addition, our procedure was suitable for detecting intracellular organelles, such as late endosomes that were recognized by anti-RAB7 antibody (Fig. S1C), and cell components such as MT1-MMP, a transmembrane metalloproteinase expressed at the basal surface of luminal cells (Fig. S1D) (Rosse et al., 2014). However, we also noticed some limitations of this method (Table 1). Noticeably, some primary antibodies successfully used in classical immunohistological studies, such as antibodies against GM130, type I and type IV collagen and aPKCι (also known as PRKCI) (Akhtar and Streuli, 2013; Lodillinsky et al., 2016; Rosse et al., 2014), did not produce staining using the uDISCO protocol described here (Table S3). Epitope accessibility issues following the mammary tissue fixation procedure could be responsible for the failed immunostaining.

**Table 1.**
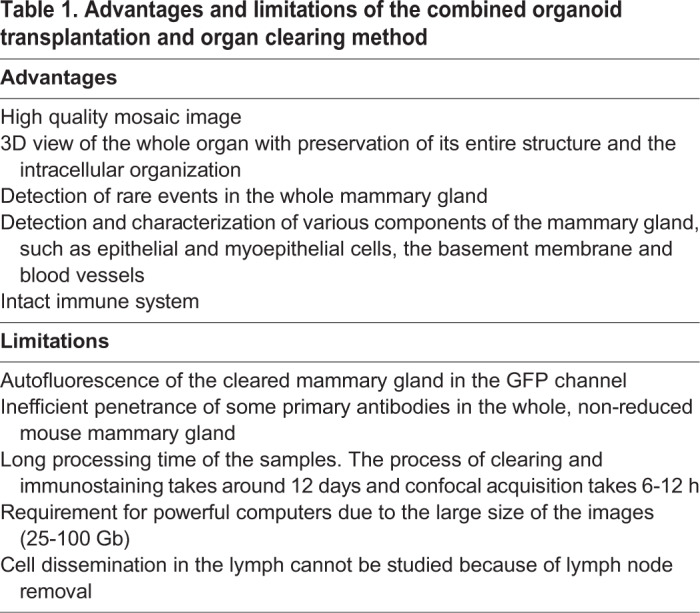
**Advantages and limitations of the combined organoid transplantation and organ clearing method**

**Fig. 2.**
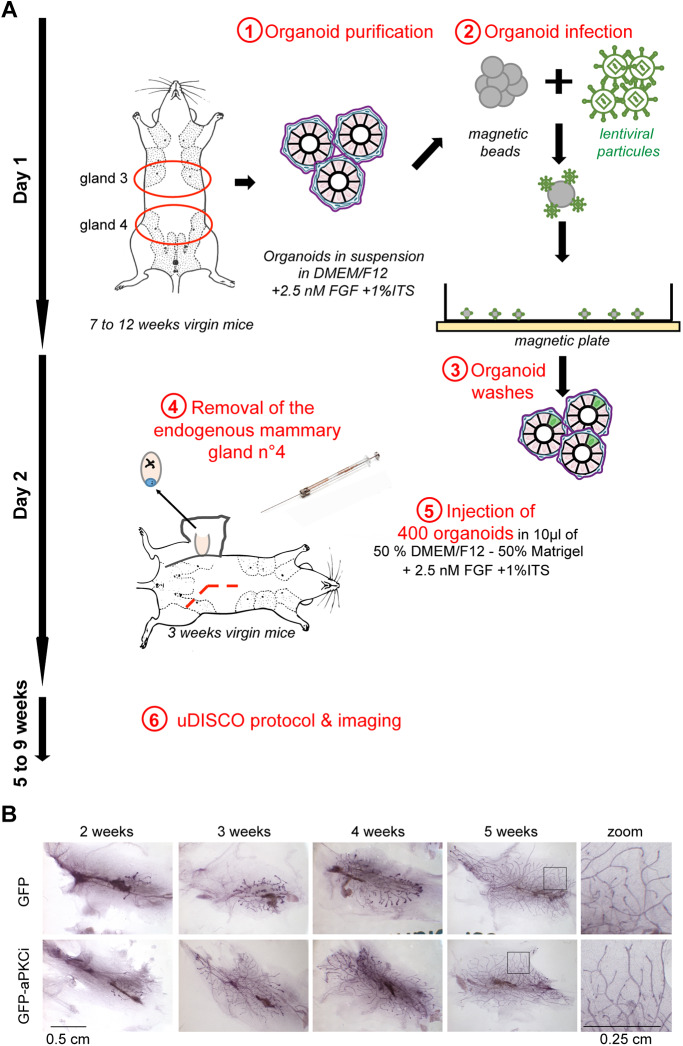
**Mouse mammary infected organoids transplantation experiment.** (A) Multistep procedure of the mouse mammary transplantation assay using lentivirally infected mammary organoids. (B) Whole-mount carmine-stained regenerated mammary glands from infected GFP^+^ or GFP-aPKCι^+^ organoids sampled at different weeks post-transplantation.

To challenge the ability of our system to identify and visualize rare cell events, we took advantage of our recently reported study in which we found that overexpression of aPKCι (aPKCι^+^) in luminal epithelial cells was sufficient to trigger basally oriented cell extrusion of aPKCι^+^ cells from the normal mammary epithelium (Villeneuve et al., 2019). Of note, aPKCι is an oncogenic protein (Fields and Regala, 2007; Parker et al., 2014; Regala et al., 2005) that is overexpressed in many carcinomas. In addition, we and others reported that aPKCι overexpression is associated with bad prognosis or outcome in breast cancer (Awadelkarim et al., 2012; Rosse et al., 2014). Thus, although not proven, it is possible that once they have reached the stroma, basally extruded epithelial aPKCι^+^ cells could potentially invade the surrounding tissue structures. Using the uDISCO technique, we aimed at visualizing isolated GFP^+^ or GFP-aPKCι^+^ cells in regenerated mammary gland mainly composed of uninfected healthy cells. To this goal, mammary gland fragments were purified as multicellular organoids and infected with lentiviral particles at low multiplicity of infection (moi) to generate GFP or GFP-aPKCι-expressing cells at low frequency (Fig. 2A). Using this strategy, we could overexpress aPKCι in a limited number of mammary luminal cells as recently shown (Villeneuve et al., 2019).

The mammary organoid purification and *ex vivo* infection steps were achieved within one day. The second day, control GFP (GFP^+^) or GFP-aPKCι (GFP-aPKCι^+^) organoids were transplanted into the cleared mammary fat pad of a 3-week-old host female of identical FVB genetic background as the donor mouse (Fig. 2A). This *in vivo* grafting assay, originally described by Deome et al. (1959), is widely used to study developmental features of the mammary gland as well as mammary tumor initiation and progression (Teuliere et al., 2005; Macias and Hinck, 2012). To minimize interindividual variability in relation to the hormonal cycle and the number of animals, control GFP^+^ and mutant GFP-aPKCι^+^ organoids were co-laterally injected into the fat pad of the same recipient mouse. After five weeks, grafted organoids successfully regenerated a mammary ductal network, typical of a virgin mouse. We did not observe any visible morphological differences between the mammary epithelial trees derived from control GFP^+^ or GFP-aPKCι-expressing organoids (Fig. 2B).

**Fig. 3.**
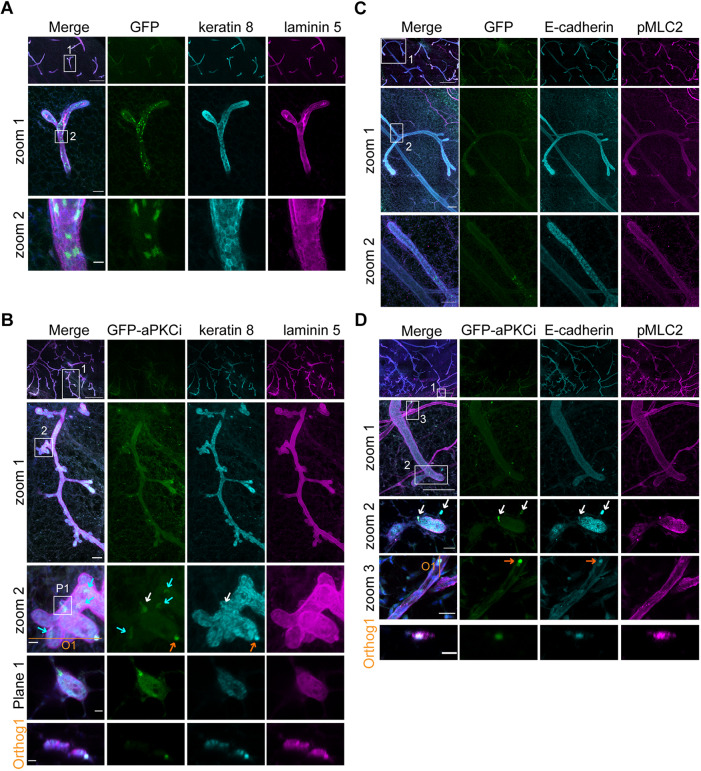
**Combination of mouse mammary organoid transplantation and organ clearing allows detection of aPKCι^+^ luminal epithelial cells breaching the basement membrane and disseminating into the surrounding stroma.** (A,B) Confocal images of regenerated mammary glands from GFP^+^ (A) or GFP-aPKCι^+^ (B) infected mouse organoids. The basement membrane and epithelial cells were stained with anti-laminin 5 (magenta) and anti-keratin 8 (cyan) antibodies, respectively. (A) Upper row shows mosaic reconstruction of z-projection of 93 confocal planes (186 µm thickness); zooms 1 and 2 show z-projection of 15 confocal planes (30 µm thickness). Numbered boxes indicate regions shown in zoom images. Scale bars: 500 µm (upper row), 50 µm (zoom 1) and 10 µm (zoom 2). (B) Upper row, zoom 1 and zoom 2 show mosaic reconstruction of z-projections of 183 confocal planes (366 µm thickness). Plane 1 shows confocal image (one confocal plane of zoom 2, in the region indicated by box P1). Orthog. 1 shows orthogonal projection corresponding to the orange line in zoom 2. The blue arrows indicate GFP-aPKCι^+^ luminal cells present in the luminal cell layer. White and the orange arrows indicate GFP-aPKCι^+^ luminal cells breaching the basement membrane. Scale bars: 500 µm (upper row), 50 µm (zoom 1) and 10 µm (zoom 2, plane 1 and orthog. 1). (C,D) Mosaic reconstruction of confocal images acquired 10 weeks after transplantation of regenerated mammary glands from GFP^+^ (C) or GFP-aPKCι^+^ (D) infected mouse organoids. Samples were stained with anti-pMLC2 (magenta) and anti-E-cadherin (cyan) antibodies. The pMLC2 staining allows the detection of the myoepithelial cell layer and the blood vessel. E-cadherin staining allows detection of the mammary luminal cells. (C) Z-projection of 152 confocal planes (304 µm thickness). Numbered boxes indicate regions shown in zoom images. Scale bars: 500 µm (upper row), 100 µm (zoom 1) and 50 µm (zoom 2). (D) Upper row and zoom 1 show z-projection of 176 confocal planes of confocal images (352 µm thickness). Zoom 2 shows z-projection of 24 confocal planes (48 µm thickness) showing GFP-aPKCι^+^ luminal epithelial cells escaping from the mammary ducts and in the stroma. Zoom 3 shows z-projection of 18 confocal planes (36 µm thickness). Orthog. 1 shows orthogonal projection (orange line O1 indicated in zoom 3). Zoom 3 and orthog. 1 show GFP-aPKCι^+^ luminal epithelial cells present in blood vessels. Scale bars: 500 µm (upper row), 100 µm (zoom 1), 20 µm (zooms 2 and 3) and 10 µm (orthog. 1).

Having established the transplantation assay for mammary organoids, we then applied the uDISCO protocol to perform imaging of the regenerating mammary epithelium. At 5-10 weeks after transplantation, intracardiac fixation of the host mice was performed as described in the Materials and Methods section (Fig. 1A), followed by double immunostaining for keratin 8 and laminin 5 (Fig. 3A,B) or phospho-myosin light chain 2 (pMLC2) and E-cadherin (Fig. 3C,D). Both keratin 8 and E-cadherin are known to be specifically expressed by luminal cells, whereas pMLC2 is a myoepithelial and endothelial cell marker. Laminin 5 is a major component of the basement membrane. The regenerated mammary gland was then cleared using the uDISCO procedure described above. Analysis of the GFP signal, together with lineage-specific markers, showed that mammary ducts contained only a few GFP^+^ or GFP-aPKCι^+^ cells surrounded by mostly wild-type GFP-negative luminal cells (Fig. 3; Movies 1-3).

Similar to the normal mammary gland, a significant reduction in total cell surface area (about 45%) and nucleus surface area (about 35%) (Fig. S2A,B) was observed without a significant reduction in the ratio of nucleus surface area to total cell surface area (Fig. S2C). All GFP^+^ epithelial cells were localized into the ducts (Fig. 3A), whereas GFP-aPKCι^+^ cells, positive for keratin 8, were able to breach the basement membrane (Fig. 3B; Movie 2). These data, which confirm our previous report (Villeneuve et al., 2019), show that GFP-aPKCι^+^ cells could leave the luminal epithelial cell layer of the duct by transmigrating through the basement membrane (Fig. 2A,B; Villeneuve et al., 2019). Similarly, using pMLC2 in combination with E-cadherin staining, we found that all GFP^+^ epithelial cells were localized in the ducts (Fig. 3C; Movie 3), whereas we could detect some GFP-aPKCι^+^ cells, positive for E-cadherin, which were able to breach the pMLC2-positive myoepithelial cell layer (Fig. 3D; Fig. S3B, Movie 4). As we previously showed (Villeneuve et al., 2019), basally extruded ‘escaping’ GFP-aPKCι^+^ cells were positive for the luminal markers keratin 8 and E-cadherin, suggesting that they did not undergo a full epithelial-to-mesenchymal transition (EMT) program upon migration. It would be interesting to further characterize the gene expression profile of these aPKCι^+^ invading epithelial cells and to investigate their migratory (i.e. mesenchymal or amoeboid, possibly collective) modes of migration *in vivo* (Cheung et al., 2016; Gligorijevic et al., 2014; Padmanaban et al., 2019; Te Boekhorst et al., 2016).

## DISCUSSION

Here, we show a novel imaging-based method for studying the onset of mammary gland oncogenesis, allowing whole-organ imaging analysis with (sub)cellular resolution while preserving the tissue context. Through combined use of infected organoid transplantation and uDISCO clearing, we were able to detect small numbers of cells in the whole organ and rare cellular events occurring *in vivo* (Fig. 3). However, although this technique is compatible with immunodetection of endogenous and tagged proteins, we noticed that some level of autofluorescence in the GFP channel could disturb the visualization of weak signals. In this respect, it might be useful to implement and adapt the protocol recently designed for decreasing autofluorescence in the brain (Li et al., 2018). A summary of the main advantages and limitations of the present method is provided in Table 1.

Using this analytical pipeline, we confirmed that aPKCι overexpression can promote basal extrusion of mammary luminal epithelial cells and cell invasion into the stroma. These data point to a potential path for cell dissemination without or before primary tumor detection, a metastasis mechanism reported in previous works (Harper et al., 2016; Hosseini et al., 2016). Most probably, aPKCι overexpression is not sufficient to trigger the full metastatic cascade. Invading cell(s) have to adapt to different microenvironments, survive and proliferate, suggesting that other oncogenic events are necessary for the acquisition of cell survival and proliferation properties.

In a more general context, we believe that this method is useful for analyzing tumor progression in various genetically modified mouse models, after or without mammary organoid transplantation (Harper et al., 2016; Hosseini et al., 2016; Padmanaban et al., 2019). We anticipate that this method can allow a more thorough investigation of the main features of primary tumor and metastasis sites, such as the lung and brain. The possibility of using various antibodies permits better characterization of EMT traits and mesenchymal/amoeboid motion and collective patterns of the invading cells during the different stages of tumor progression *in vivo*. Moreover, this pipeline can be applied to study mammary gland development, for example to investigate the ability of genetically modified stem cells to regenerate the mammary epithelium or the cellular crosstalk between mammary stroma and the epithelium.

## MATERIALS AND METHODS

### Ethical approval

Animal care and use for this study were performed in accordance with the recommendations of the European Community (2010/63/UE) for the care and use of laboratory animals. Experimental procedures were specifically approved by the ethics committee of the Institut Curie CEEA-IC #118 (CEEA-IC 2017-013) in compliance with international guidelines.

### Isolation of the mammary epithelium and generation of organoids

The mammary epithelium isolation protocol was similar to published methods (Ewald, 2013; Nguyen-Ngoc et al., 2012). Briefly, the two #3 and #4 glands on both sides of the mice were collected and lymphatic ganglions from #4 glands were removed. Then, the mammary glands were cut with a scalpel about 50 times and the small pieces transferred into 25 ml of a collagenase solution (Table S1) (volume used for the mammary glands of a maximum of two mice). The collagenase solution containing the glands was incubated at 37°C with shaking at 120 rpm for 1 h and a manual shake for 30 min.

After this step, all the tubes and tips were coated with PBS containing 0.3% BSA. The collagenase solution containing mammary gland fragments was transferred into a glass tube (tube #1) and centrifuged at 500×***g*** for 10 min at 25°C. Three layers were obtained in the tube: a fatty layer at the top, an aqueous layer in the middle and a basal layer containing the epithelial material at the bottom. The fatty layer was transferred into a second tube (tube #2) containing 25 ml collagenase solution and incubated for 20 min at 37°C and 120 rpm.

Meanwhile, tube #1 was centrifuged at 500×***g*** for 10 min at 25°C and the pellet resuspended in 10 ml of organoid medium without ITS (Table S1).

At the end of 20 min, tube #2 was centrifuged at 500×***g*** in a glass tube for 10 min at 25°C. The pellet was resuspended in 2 ml of organoid medium without ITS before being transferred into tube #1. Tube #1 was then centrifuged at 500×***g*** for 10 min at 25°C. The pellet was resuspended in 4 ml of DNAse solution (Table S1) and mixed by hand for 2-5 min at room temperature (RT). Then, 6 ml of organoid medium without ITS was added and the tube centrifuged at 500 ***g*** for 10 min at 25°C. Purification of the pellet of organoids was performed, consisting of two rounds of 2 min centrifugation at 500×***g*** for 10 min followed by three pulses of centrifugation at 500×***g*** in 10 ml of organoid medium.

The last pellet was resuspended in the appropriate volume of organoid medium supplemented with 1% ITS and 2.5 nM FGF (about 600-800 organoids/ml). Aliquots of 700 μl/well were distributed in non-adherent 24-well plates.

### Low-efficiency lentiviral infection of organoids

#### Production of lentiviral particles

##### One day before transfection

HEK 293T cells were grown in 25 cm^2^ culture flasks on appropriate medium (Table S1) at 37°C and 5% CO_2_ (two flasks per condition) to obtain 60-70% confluence on the day of transfection.

##### Day of transfection

The medium was changed 2 h before transfection. A mix of 400 μl of OptiMem medium and 18 μl of GeneJuice was made and mixed by vortex (tube A). The mix was incubated at room temperature for 5 min. In parallel, 0.9 μg pCMV VSVg, 2.1 μg psPAX 2 and 3 μg of vector containing the protein to express were gently mixed with 400 μl of OptiMem medium by up and down pipetting (tube B).

The contents of tubes A and B were mixed up and down in a total volume of 800 μl. The final mix was incubated at room temperature for 20 min. For each flask, 400 μl of the mix was added drop by drop. Cells were incubated for 60 h at 37°C and 5% CO_2_.

#### Concentration of lentiviral particles

Supernatant containing the lentiviral particles was sampled and filtered with a 0.45 μm syringe filter. After filtration, the supernatant was concentrated with Lenti-X concentrator (one volume of Lenti-X concentrator for three volumes of supernatant) and the mix incubated for 2 h at 4°C. Then, the mix was centrifuged at 1500×***g*** for 45 min at 4°C. The pellet was resuspended in organoid medium to concentrate the viral particles solution 50 times. The solution was aliquoted and frozen at −80°C until the experiment was performed. Free-thaw cycles should be avoided.

#### Infection of the organoids

Magnetic beads were added to the viral particle solution (Table S2) (one tenth of the volume of virus particle solution) and incubated for 20-25 min at RT. Then, the mix of magnetic beads and virus particle solution was added to organoids plated on non-adherent 24-well plates (55 µl of the mix for one well). The non-adherent 24-well plates were put on a magnet for 1 h 30 min at 37°C at 5% CO_2_. Then, the magnet was removed and the plates incubated at 37°C and 5% CO_2_ for 24 h.

Note 1: During manipulation, all plastics were coated with PBS containing 0.3% BSA.

#### Preparation of the organoids for transplantation

Organoids were transferred into a 50 ml falcon tube and washed three times in 5 ml of PBS containing 0.3% BSA, with three centrifugation cycles of 10 min at 500×***g*** and RT. Organoids were resuspended at a concentration of 400 organoids per 10 µl transplantation medium (Table S1).

### Transplantation

Between four and six animals per experiment were transplanted in order to obtain significant results per lot. The entire transplant procedure was performed under general anesthesia. The anesthetic solution used contained 10 mg/ml ketamine (also an analgesic), 1 mg/ml xylazine and 1 mg/ml flunitrazepam (benzodiazepine, which ensures a loss of consciousness and a peaceful awakening) (Table S2). This solution was diluted by two in PBS and 100 µl injected intraperitoneally per 10 g of mouse. The anesthetized mice were placed on a warm cushion throughout the operation to maintain their body temperature at 37°C. Before the operation, 200 µl of physiological serum was subcutaneously injected to hydrate the mice. During surgery, the legs were held by a flexible adhesive that did not impede blood circulation and mice were depilated on the operated area. The skin of the abdomen was incised (an intact peritoneum was preserved) on 2 cm. The vessels were cauterized with a cauterizer and the original gland was eliminated. Then, 10 µl of cells (equivalent to about 400 organoids resuspended in transplantation medium) (Table S1) was injected using an ultrafine Hamilton syringe (25 µl Hamilton 702 RN). After surgery, the skin was sewn using resorbable surgical thread to avoid discomfort and infection. Postoperatively, the anesthetized mice were placed on a hot plate to avoid hypothermia. Upon waking, mice were placed in a new cage with water and food on demand. During the first two weeks, hydrogel cups with ground food were placed in cages to facilitate food access for mice. The mice were monitored every day for the first 4 days after transplantation, then once a week. The skin was quickly (one week approximately) healed and mice were not restricted in their movements.

### 3D tissue immunofluorescence

Mice were sacrificed at the latest 5-10 weeks after transplantation and the #4 mammary glands collected. For mammary gland immunofluorescence, without clearing and reduction, tissue immunofluorescence was performed as described (Villeneuve et al., 2019).

Note 2: The fixed and immunostained tissues could not be kept for more than 2 weeks. Freezing the sample would impact the structure of the gland and immunostaining.

### Histology of mouse regenerated mammary gland tissue

Whole-mount carmine staining was performed as described previously (Teuliere et al., 2005).

### Intracardiac fixation

The animals were deeply anesthetized with ketamine (80-100 mg/ml; Table S2) mixed with the analgesic xylasin (5-10 mg/ml; Table S2) in PBS. The mixture was injected intraperitoneally at a concentration of 50 μl per 10 g of mouse weight. The efficiency of the anesthesia was checked by pinching the mice, and the procedure was started if no muscle response was detected. An incision was made from the lower abdomen to the top of the thorax and the rib cage was opened. The needle of the peristaltic pump was inserted into the left ventricle of the heart and the vein returning the blood to the right atrium was severed. A solution of PBS was perfused, followed by PBS containing 4% paraformaldehyde (30 ml per mice).

### Staining uDISCO

#### Whole gland staining

The whole gland was fixed for 3-4 h in PBS containing 4% PFA at 4°C. The staining protocol was adapted from a published method (Krndija et al., 2019). Briefly, the gland was permeabilized using PBS containing 1% Triton X-100 overnight at RT under shaking. Directly after permeabilization, the gland was incubated in blocking solution for 8 h in PBS containing 0.2% Triton X-100, 1% BSA and 3% FBS. Then, the gland was incubated for at least 2 days at RT in 300-500 µl of primary antibody diluted to an adequate concentration (Table S3) in PBS containing 0.2% Triton X-100. Primary antibodies that did not work using the described protocol are mentioned in Table S3. The gland was washed three times during the day with PBS containing 0.2% Triton X-100. The gland was then incubated with secondary antibody diluted in PBS containing 0.2% Triton X-100 at RT for at least 2 days (Table S3 for the dilutions). The gland was washed three times during the day with PBS containing 0.2% Triton X-100.

#### Tissue clearing using the uDISCO technique

The samples were washed in 0.1 M PBS for 5 min before clearing. All incubation steps were performed in a chemical hood with gentle rotations or shaking. The samples were covered with aluminium foil to protect them from light. The fixed samples were incubated at 35°C overnight in tert-butanol diluted in distilled water at 30 vol%, 4 h in 50 vol%, 4 h in 70 vol%, overnight in 80 vol%, 4 h in 90 vol%, 4 h in 96 vol%, 4 h in 100% tert-butanol, and then in dichloromethane for 45-60 min at RT. Finally, the samples were incubated in BABB-D15 composed of BABB (benzyl alcohol and benzyl benzoate at a ratio of 1:2) plus diphenyl ether (ratio 1:15 BABB) and 0.4% vitamin E for 2-6 h until the samples became optically transparent and kept in this solution.

Note 3: If samples are not immediately cleared after immunostaining, they should be stored at 4°C in PBS containing 0.2% Triton X-100.

### Imaging

Confocal images of cleared samples or mammary tissues were performed on a CLSM Leica TCS SP8 inverted microscope equipped with hybrid detectors HyD. Depending on the magnification, several objectives were used (20× PL ApoDry 0.75 NA, 40× PL Apo oil 1.25 NA objectives). Mosaic images were reconstructed using the Leica SP8 software. Images were processed using ImageJ software.

### Statistical analysis

All error bars represent the standard error of the mean (s.e.m.). The statistical test used for each graph is mentioned in the legend of the graph. Statistical significance was defined as ***P<0.001. All calculations and plots were performed and generated using GraphPad Prism software.

## Supplementary Material

Supplementary information

Reviewer comments
