# Supplementary material for: A new pipeline for pathophysiological analysis of the mammary gland based on organoid transplantation and organ clearing
Source: J Cell Sci. 2020 Jun 23;133(12):jcs242495. doi: 10.1242/jcs.242495 (PMC7328142; doi:10.1242/jcs.242495)
Supplement: Supplementary information [file joces-133-242495-s1.pdf]

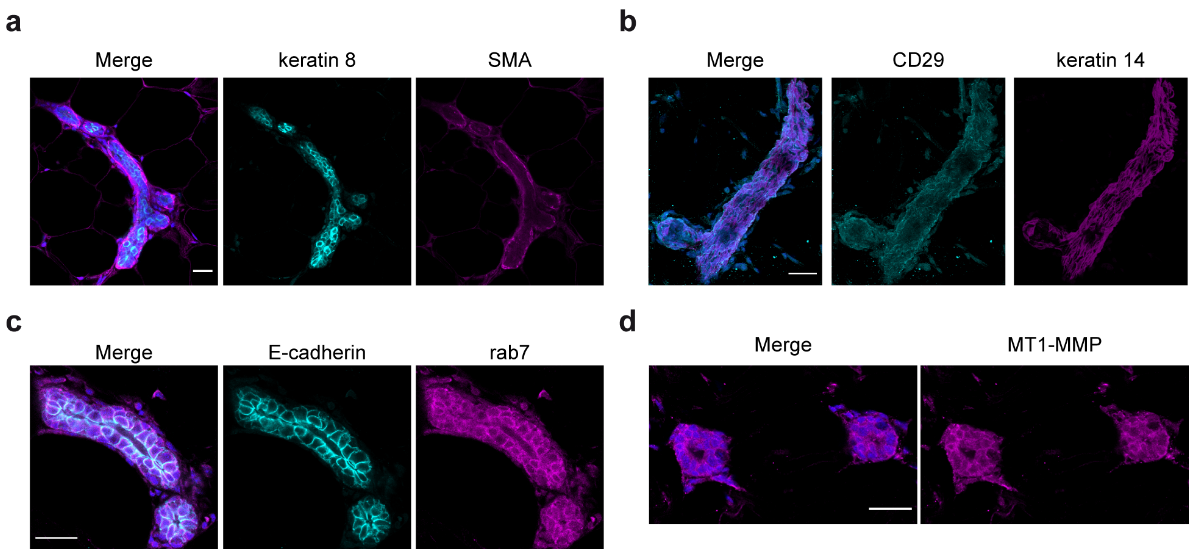

Figure S1. Examples of immunostaining of mouse mammary gland clearing using uDISCO. (a) Confocal image of mouse mammary glands stained with anti-Smooth Muscle Actin (SMA) (magenta) and anti-keratin 8 (cyan) antibodies, respectively. (b) z-projection of 10 confocal planes with 2 µm interval (20 µm total thickness) of of mouse mammary glands stained with anti-keratin14 (magenta) and anti-CD29 (cyan) antibodies, respectively. (c) Confocal image of mouse mammary glands stained with anti-rab7 (magenta) and anti-E-cadherin (cyan) antibodies, respectively. (d) Confocal image of mouse mammary glands stained with anti-MT1-MMP antibody. Scale bars, 20 µm.

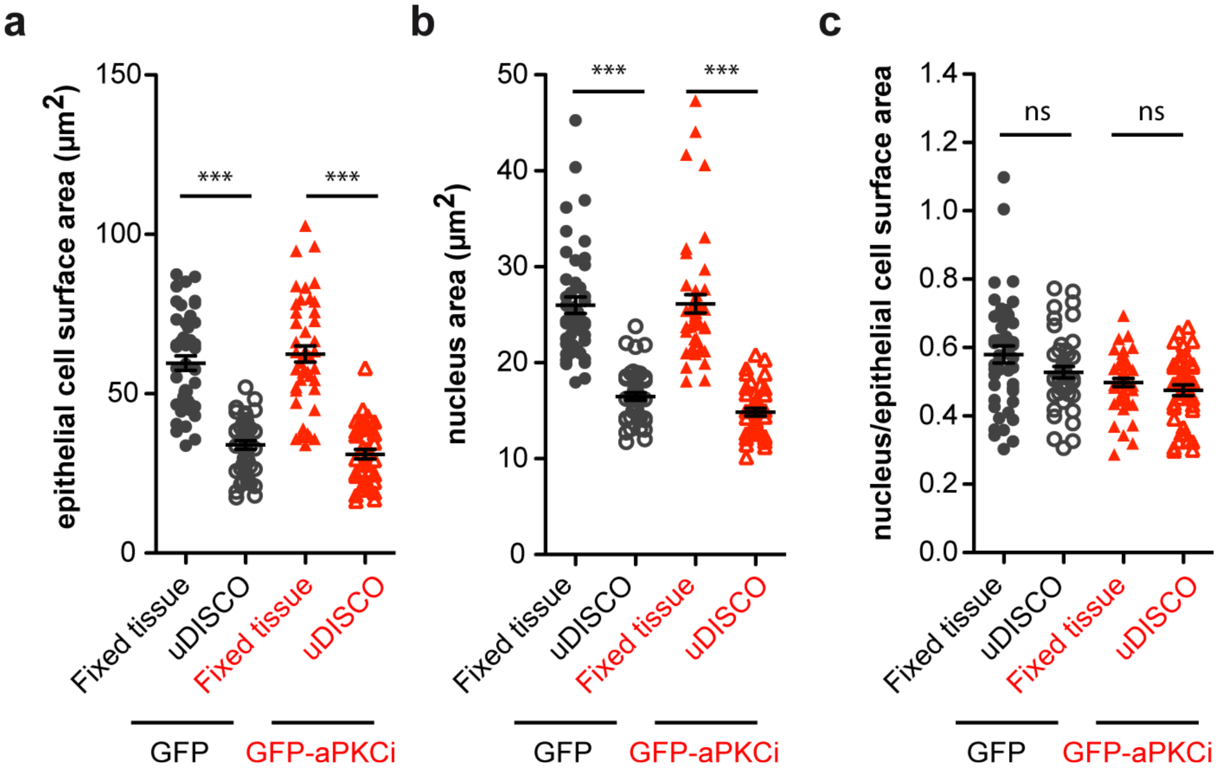

Figure S2. Quantification of epithelial cell surface and nucleus area of fixed tissue (fixed tissue) or fixed tissue followed by uDISCO treatment (uDISCO) from regenerated mammary glands from GFP^{+} or GFP-aPKCi^{+} organoids. (a) Quantification of epithelial cell surface area. The epithelial cell surface and the nucleus area were manually measured in the cell medium confocal plane, respectively using an E-cadherin and a DAPI staining. A Kruskal-Wallis test was performed (***p < 0.001). (b) Quantification of epithelial nucleus surface area, a Kruskal-Wallis test was performed (***p < 0.001). (c) Quantification of the ratio between epithelial nucleus surface area and the epithelial cell surface area. A Kruskal-Wallis test was performed (ns, nonsignificant). (a-c) For "GFP-fixed tissue", "GFP-uDISCO", "GFP-aPKCi-fixed tissue" conditions, 45 cells from 3 independent experiments were quantified. For "GFP-aPKCi-uDISCO" condition, 41 cells from 3 independent experiments were quantified.

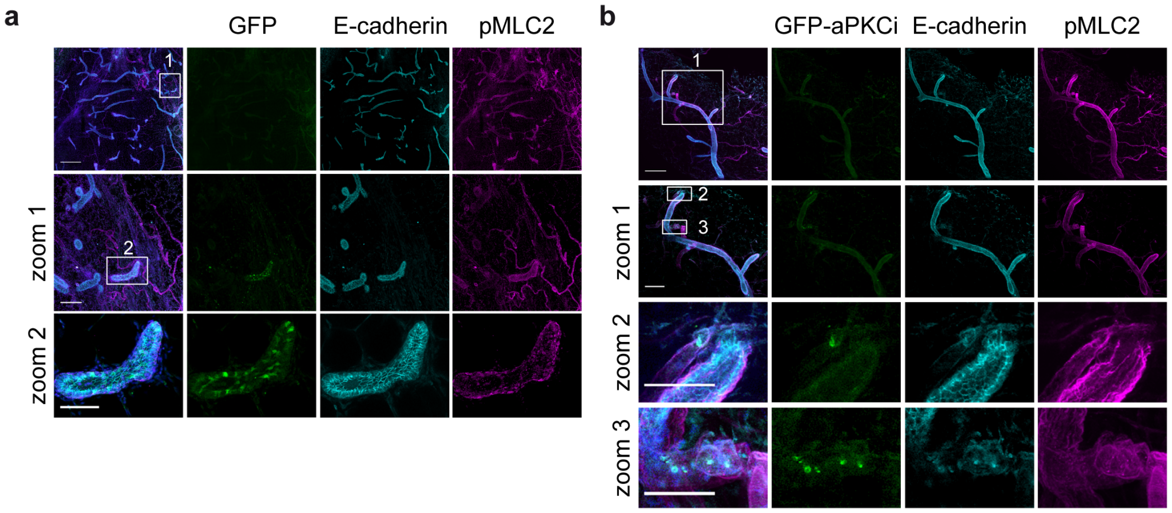

Figure S3. Combination of Organ clearing and mouse mammary organoids transplantation allows the detection of aPKCi$^+$ luminal epithelial cells breaching the myoepithelial cell layer and disseminating into the mammary stroma. Six weeks post transplantation regenerated mammary glands were stained with anti-pMLC2 (magenta) and anti-E-cadherin (cyan) antibodies, respectively. The pMLC2 staining allows the detection of the myoepithelial cell layer and the blood vessel. The E-cadherin staining allows the detection of the mammary luminal epithelial cells. (a) Upper line panels: z-projection of mosaic reconstruction of 102 confocal planes (204 µm thickness) of regenerated mammary glands from GFP$^+$ infected mouse organoids. Scale bar, 500 µm. Zoom 1: z-projection of 70 confocal planes (140 µm thickness). Scale bar, 100 µm. Zoom 2: z-projection of 7 confocal planes (14 µm thickness). Scale bar, 50µm. (b) Upper line panels and zoom1: z-projection of mosaic reconstruction of 97 confocal planes (194 µm thickness) of regenerated mammary glands from GFP-aPKCi$^+$ infected mouse organoids. Scale bar, 500 µm. Zoom1: z-projection of 37 confocal planes (74 µm thickness). Scale bar, 100 µm. Zoom 2&3: z-projection of 10 confocal planes (20 µm thickness) of regenerated mammary glands from GFP-aPKCi$^+$ infected mouse organoids showing GFP-aPKCi$^+$ luminal epithelial cells escaping from the mammary ducts and in the stroma. Scale bars, 20 µm.

Table S1. Medium composition

Media	Composition
Collagenase solution	DMEM/F12 + 5% FBS + 12% Trypsin 10X + 25 mg Gentamicin + 2.5 mg human insulin + 50 µg Collagenase
DNase solution	DMEM/F12 + 1% Glutamine +1% Penicillin/streptomycin + 4 U/mL of DNase I
Organoid medium (during purification)	DMEM/F12 + 1% Glutamine + 1% Penicillin/streptomycin
Organoid medium (during infection)	DMEM/F12 + 1% Glutamine + 1% Penicillin/streptomycin + 1% ITS + 2.5 nM FGF
Transplantation medium	50% organoid medium + 50% matrigel + 2.5 nM FGF
Hek293T- lentiX medium	DMEM High Glucose GlutaMAX + 10% FBS + 1% Penicillin/streptomycin

Journal of Cell Science • Supplementary information

Table S2. Reagents used for this study

Organoids purification		
**Reagent**	**Reference**	**Manufacturer**
ITS (human insulin, human transferrin, sodium selenite)	I3146-5ml	Sigma
Glutamine	25030024	Gibco
Penicillin/Streptomycin	15140122	Gibco
Trypsin 10X	15400054	Gibco
DMEM/F12	11039-047	Gibco
Human Insulin	I9278-5ml	Sigma
Gentamicin	15750-037	Gibco
HBSS	14025-050	Gibco
Collagenase	10103578001	Sigma
DNase I	D4263	Sigma
FGF	PMG0034	Thermo Fisher
Corning® Costar® Ultra-Low Attachment Multiple Well Plate 24 wells	CLS3473-24EA	Sigma
**Organoids infection**		
**Reagent**	**Reference**	**Manufacturer**
ViroMag R/L Transduction Reagent	RL40200	Oz Biosciences
**Lentiviral particles preparation**		
**Product**	**Reference**	**Manufacturer**
DMEM High Glucose GlutaMax	61965-059	Life technologie
Lenti-X 293T Cell line	632180	Ozyme
Lenti-X Concentrator	631231	Ozyme
GeneJuice® Transfection Reagent	70967-3	Merck
**Animals care / surgery**		
**Reagent**	**Reference**	**Manufacturer**
Syringe 25 µl Hamilton 702 RN	74329	Dutscher
Gemini Cautery Kit	72-6067	Harvard Apparatus
Ketamine	Imalgen ® 1000	Merial
Xylazine	Rompun ® 2%	Bayer
Rod 16-R 21 Kgy	Rod16-R	Genobios
**uDISCO clarification**		
**Reagent**	**Reference**	**Manufacturer**
benzyl benzoate	B6630-500ML	Sigma
benzyl alcohol	24122-1L-D	Sigma
tert-butanol	360538-1L-D	Sigma
DICHLOROMETHANE, ANHYDROUS, >=99.8%, CO	270997-100ML	Sigma
Diphenyl ether, 99%	A15791.30	VWR
DL-alpha-Tocopherol, 97+%	A17039.18	VWR

Journal of Cell Science • Supplementary information

Table S3. Antibodies references and dilutions used for this study

Primary antibodies	Species	Dilution	Reference	Manufacturer
CD29 (clone 9EG7)	Rat	1/100	550531	BD Biosciences
E-cadherin (ECCD-2)	Rat	1/200	13-1900	Invitrogen
GFP	Chicken	1/200	ab13970	Abcam
keratin 5	Rabbit	1/200	BLE905501	Ozyme
keratin 8	Rat	1/200	TROMA-I	DSHB
keratin 14	Rabbit	1/200	BLE905301	Ozyme
laminin 5	Rabbit	1/2000	Gift from M. Aumeilley (Cologne)	
Phospho-Myosin Light Chain 2 (Ser19)	Rabbit	1/50	3671S	Ozyme
MT1-MMP	Rabbit	1/100	ab51074	Abcam
rab7 (clone D95F2)	Rabbit	1/75	9367	Cell Signaling Tech
Smooth Muscle Actin	Mouse	1/100	M0851	Dako
ZO-1 clone R40.76	Rat	1/100	MABT11	Millipore
**Secondary antibodies**		**Dilution**	**Reference**	**Manufacturer**
IgG-chicken 488	Goat	1/200	A11039	Life Technologies
IgG-rabbit Cy3	Donkey	1/200	711-165-152	Jackson Immunoresearch
IgG-rabbit Alexa 647	Donkey	1/200	A31573	Invitrogen
IgG-rat Alexa Fluor 647	Goat	1/200	A21247	Molecular Probes
**Tested primary antibodies - not working**		**Dilution**	**Reference**	**Manufacturer**
Type I collagen	Mouse	1/200	C2456	Sigma
Type IV collagen	Rabbit	1/200	ab19808	Abcam
GM130	Mouse	1/100	610823	BD Biosciences
aPKCi	Mouse	1/100	610175	BD Biosciences

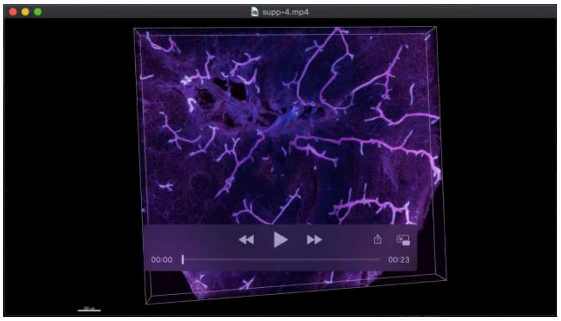

Movie 1. 3D reconstruction of FVB mouse mammary gland 4 performed on Imaris software. The movie corresponds to the z-stack shown in Fig. 1d of 145 confocal planes (290 µm thickness) of cleared mouse gland 4 showing the localization of laminin 5 (in magenta) and keratin 8 (in cyan). Z-stack images were captured at 2 µm interval.

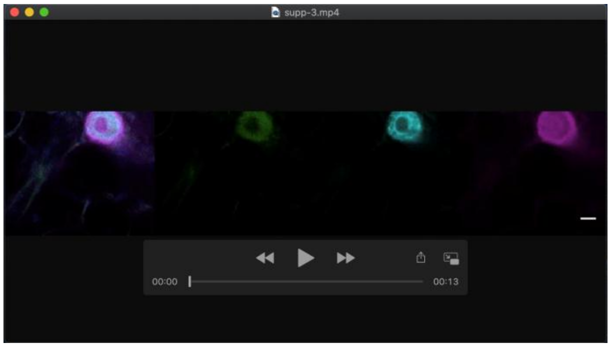

Movie 2. Detection of GFP-aPKCi^{+} luminal epithelial cells breaching the basement membrane and disseminating into the surrounding stroma. The movie corresponds to the z-stack shown in Fig. 3b – zoom 2 showing the localization of laminin5 (in magenta) and keratin8 (in cyan). Z-stack images were captured at 2 µm interval.

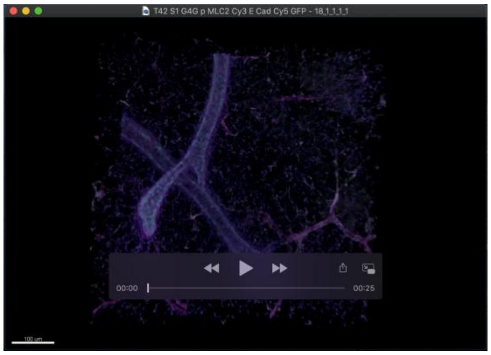

Movie 3. Detection of GFP$^+$ luminal epithelial cells of regenerated mammary glands from GFP$^+$ infected mouse organoids: 3D reconstruction performed on Imaris software corresponding to the figure 3c of 152 confocal planes (304 µm thickness). Z-stack images were captured at 2 µm interval.

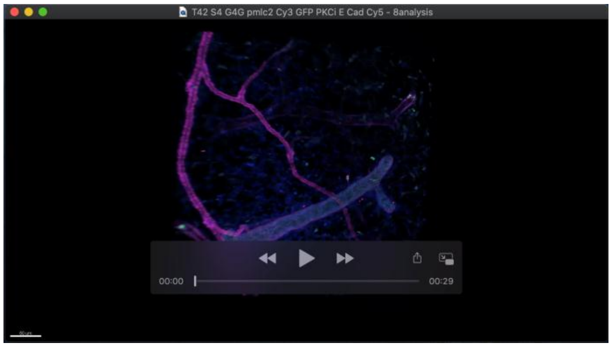

Movie 4. Detection of GFP-aPKCi$^+$ luminal epithelial cells of regenerated mammary glands from GFP-aPKCi$^+$ infected mouse organoids: 3D reconstruction performed on Imaris software corresponding to the figure 3d of 176 confocal planes (352 µm thickness). Z-stack images were captured at 2 µm interval.

Journal of Cell Science • Supplementary information
