# Supplementary material for: A new pipeline for pathophysiological analysis of the mammary gland based on organoid transplantation and organ clearing
Source: J Cell Sci. 2020 Jun 23;133(12):jcs242495. doi: 10.1242/jcs.242495 (PMC7328142; doi:10.1242/jcs.242495)
Supplement: Reviewer comments [file joces-133-242495_review_history.pdf]

A new pipeline for pathophysiological analysis of the mammary gland based on organoid transplantation and organ clearing

Emilie Lagoutte, Clémentine Villeneuve, Vincent Fraisier, Denis Krndija, Marie-Ange Deugnier, Philippe Chavrier and Carine Rossé

DOI: 10.1242/jcs.242495

Editor: Andrew Ewald

Review timeline

Original submission:	2 December 2019
Editorial decision:	20 January 2020
First revision received:	23 April 2020
Accepted:	13 May 2020

Original submission

First decision letter

MS ID#: JOCES/2019/242495

MS TITLE: A new pipeline for the study of the onset of mammary gland oncogenesis based on mammary organoid transplantation and organ clearing

AUTHORS: Emilie Lagoutte, Clementine Villeneuve, Vincent Fraisier, Denis Krndija, Marie-Ange Deugnier, Philippe Chavrier, and Carine Rosse
ARTICLE TYPE: Tools and Resources

We have now reached a decision on the above manuscript.

To see the reviewers' reports and a copy of this decision letter, please go to: https://submit-jcs.biologists.org and click on the 'Manuscripts with Decisions' queue in the Author Area. (Corresponding author only has access to reviews.)

As you will see, the reviewers share enthusiasm for the approach but raise a number of substantial criticisms that prevent me from accepting the paper at this stage. The essential issue is that the manuscript at present is reading partway between a methods and a research paper. I understand how that could happen, given our instructions to authors. The challenge here is a bit how to think about clearing in the mammary gland; as the reviewers point out, clearing isn't a new method, though its application to the mammary gland seems useful. I could see multiple ways to focus revisions of the manuscript, ranging from deepening the molecular analysis of metastasis mechanisms with iDISCO as a key technique or streamlining of the biology (and rewriting of the conclusions) along with a deepening of the manuscript as a technical resource by demonstrating more utility of the method and validating more antibodies. The reviewers suggest that a revised version might prove acceptable, if you can address their concerns. If you think that you can deal satisfactorily with the criticisms on revision, I would be pleased to see a revised manuscript. We would then return it to the reviewers. If you would like to propose a revision plan for my review, that is fine, though not required.

Please ensure that you clearly highlight all changes made in the revised manuscript. Please avoid using 'Tracked changes' in Word files as these are lost in PDF conversion.

© 2020. Published by The Company of Biologists under the terms of the Creative Commons Attribution License (https://creativecommons.org/licenses/by/4.0/).

I should be grateful if you would also provide a point-by-point response detailing how you have dealt with the points raised by the reviewers in the 'Response to Reviewers' box. Please attend to all of the reviewers' comments. If you do not agree with any of their criticisms or suggestions please explain clearly why this is so.

Reviewer 1

Advance summary and potential significance to field

In this manuscript, Lagoutte et al., reported a new method to clarify mammary gland tissue for 3D immunofluorescence imaging. They applied this approach to transplanted mammary cell organoids, and examined localization of cells transfected with atypical protein kinase C iota (aPKCi+). They claimed that compared to control cells, aPKCi+ cells can breach through the basement membrane and appear in stroma. This was interpreted as early step of metastasis.
Overall, this approach is interesting and potentially useful for many purposes. However, the conclusions drawn from aPKCi+ data are not supported by the data. The relevance of the observation remains to be established for neither tumorigenesis nor metastasis. The single aPKCi+ cells observed in Figure 3d seem to be anecdotal. The cellular states and fates are unclear. Without longitudinal data showing that the tumorigenesis and metastasis do originate from aPKCi+ cells, the statements regarding aPKCi+ cells are unsubstantiated.

Comments for the author

My recommendation is to re-interpret the data, tone down the biological conclusions regarding tumorigenesis and metastasis, and elaborate and demonstrate the methodological novelty more thoroughly.

Reviewer 2

Advance summary and potential significance to field

Lagoutte et al. combine an implantation procedure of genetically modified organoids into the cleared mouse mammary fat with an adaptation of the uDISCO clearing method to perform 3D mammary gland microscopy with cellular resolution. As an interesting application, large-volume analysis of detached single cells after ectopic expression of GFP-aPKCi is clearly demonstrated. Both clearing and organoid implantation are not novel. However, the in vivo evidence of aPKCi in mediating single cell detachment from otherwise intact mammary glands is an compelling follow-up to recent in vitro work from the same group showing a role of aPKCi in mammary epithelial cell extrusion.

As is stands right now, a technological resource, with some technical controls added, this work might fit the scope of Disease Models and Mechanisms. For publication in the J. Cell Sci., deeper insight into the process of single cell dissemination and the fate of escaping cells should be provided.

Comments for the author

1. The comparison of grafted mammary glands in Fig. 2B should show that the basement membrane is fully developed, e.g., by staining for collagen IV. The authors show laminin-5 (in Fig. 3), however this is not sufficient to detail structural heterogeneity. Collagen IV is the most relevant backbone of a completely developed, mature basement membrane. Correct basement membrane formation is critical to secure that early single cell evasion is not caused by a general defect of barrier formation by a defective basement membrane poor cell anchorage and/or epithelial polarization.

2. Efforts should be undertaken to show the individualized cells with higher resolution. Can they be classified as “Amoeboid”, in line with previous work from the Condeelis group.

© 2020. Published by The Company of Biologists under the terms of the Creative Commons Attribution License (https://creativecommons.org/licenses/by/4.0/).

3. The frequency of individualized cells, their origin (terminal end buds ducts etc.) and more detail on the time course of detachment should be provided. Time-gated controls are required to exclude that single cells are remnants from much earlier stages of ductal sprouting, where the basement membrane was incomplete, or caused by the wound healing response. This would rule out an artefact of the constitutive aPKCi overexpression combined with the surgery and gland reconstruction procedure.

4. Lastly, the fate of these detached cells should be shown. It is within direct reach of this approach to show whether these cells survive and end up in distant organs, because the hematogenous route is expected to be intact.
Technically, this analysis is critical to validate the suitability of the implantation model as spontaneous metastasis model. Biologically, the data would demonstrate, whether aPKCi signaling is sufficient to drive the whole metastatic cascade.

Minor points:

Fig. 2B should include zoomed images /details on intact duct and bud structure.

Fig. 3B (e.g. zoom 2) and D (zoom 3) should also show orthogonal projections to demonstrate that the individualized cells are fully detached from the strand. Single cell detachment is obvious from the sample rotations in the movies, but evidence should also be clear in the images.

First revision

Author response to reviewers' comments

Dear Dr. Rosse,

We have now reached a decision on the above manuscript.

As you will see, the reviewers share enthusiasm for the approach but raise a number of substantial criticisms that prevent me from accepting the paper at this stage. The essential issue is that the manuscript at present is reading partway between a method and a research paper. I understand how that could happen, given our instructions to authors. The challenge here is a bit how to think about clearing in the mammary gland; as the reviewers point out, clearing isn't a new method, though its application to the mammary gland seems useful. I could see multiple ways to focus revisions of the manuscript, ranging from deepening the molecular analysis of metastasis mechanisms with iDISCO as a key technique or streamlining of the biology (and rewriting of the conclusions) along with a deepening of the manuscript as a technical resource by demonstrating more utility of the method and validating more antibodies. The reviewers suggest that a revised version might prove acceptable, if you can address their concerns. If you think that you can deal satisfactorily with the criticisms on revision, I would be pleased to see a revised manuscript. We would then return it to the reviewers. If you would like to propose a revision plan for my review, that is fine, though not required.

I should be grateful if you would also provide a point-by-point response detailing how you have dealt with the points raised by the reviewers in the 'Response to Reviewers' box. Please attend to all of the reviewers' comments. If you do not agree with any of their criticisms or suggestions please explain clearly why this is so.

We are very grateful to the editor and to the reviewers for their constructive advices on how to strengthen the study in terms of results and clarity. In particular, we have now reshaped our manuscript as a technical resource by validating more antibodies and by proposing more applications for our method, as you suggested. Please find below a point-by-point response (in blue) to the referee’s comments.

© 2020. Published by The Company of Biologists under the terms of the Creative Commons Attribution License (https://creativecommons.org/licenses/by/4.0/).

Reviewer 1

Advance Summary and Potential Significance to Field:

In this manuscript, Lagoutte et al., reported a new method to clarify mammary gland tissue for 3D immunofluorescence imaging. They applied this approach to transplanted mammary cell organoids, and examined localization of cells transfected with atypical protein kinase C iota (aPKCi+). They claimed that compared to control cells, aPKCi+ cells can breach through the basement membrane and appear in stroma. This was interpreted as early step of metastasis.

Overall, this approach is interesting and potentially useful for many purposes. However, the conclusions drawn from aPKCi+ data are not supported by the data. The relevance of the observation remains to be established for neither tumorigenesis nor metastasis. The single aPKCi+ cells observed in Figure 3d seem to be anecdotal. The cellular states and fates are unclear. Without longitudinal data showing that the tumorigenesis and metastasis do originate from aPKCi+ cells, the statements regarding aPKCi+ cells are unsubstantiated.

Comments for the Author:

My recommendation is to re-interpret the data, tone down the biological conclusions regarding tumorigenesis and metastasis, and elaborate and demonstrate the methodological novelty more thoroughly.

We have now reorganized our manuscript as a methodological resource. The effect of aPKCi overexpression on early epithelial cell invasion, which was previously described in Villeneuve et al, PNAS 2019, is now presented as a proof of concept to demonstrate the ability of our developed procedure to detect rare cellular events. We also expanded our discussion on the potential use of this protocol to study tumorigenesis and metastasis process.

Reviewer 2

Advance Summary and Potential Significance to Field:

Lagoutte et al. combine an implantation procedure of genetically modified organoids into the cleared mouse mammary fat with an adaptation of the uDISCO clearing method to perform 3D mammary gland microscopy with cellular resolution. As an interesting application, large-volume analysis of detached single cells after ectopic expression of GFP-aPKCi is clearly demonstrated. Both clearing and organoid implantation are not novel. However, the in vivo evidence of aPKCi in mediating single cell detachment from otherwise intact mammary glands is an compelling follow-up to recent in vitro work from the same group showing a role of aPKCi in mammary epithelial cell extrusion.

As is stands right now, a technological resource, with some technical controls added, this work might fit the scope of Disease Models and Mechanisms. For publication in the J. Cell Sci., deeper insight into the process of single cell dissemination and the fate of escaping cells should be provided.

Comments for the Author:

1. The comparison of grafted mammary glands in Fig. 2B should show that the basement membrane is fully developed, e.g., by staining for collagen IV. The authors show laminin-5 (in Fig. 3), however this is not sufficient to detail structural heterogeneity. Collagen IV is the most relevant backbone of a completely developed, mature basement membrane. Correct basement membrane formation is critical to secure that early single cell evasion is not caused by a general defect of barrier formation by a defective basement membrane, poor cell anchorage and/or epithelial polarization.

We agree with the reviewer that it would be beneficial to better characterize the structural integrity and composition of the basement membrane. Unfortunately, we could not perform the suggested collagen IV staining, because our available anti-collagen IV antibody (Ref: Abcam ab19808) does not

© 2020. Published by The Company of Biologists under the terms of the Creative Commons Attribution License (https://creativecommons.org/licenses/by/4.0/).

work using the present procedure. Unfortunately, some primary antibodies (Table S3), which were tested and worked using a classical immunohistochemistry protocol (Akhtar and Streuli, 2013; Lodillinsky et al., 2016; Rosse et al., 2014) did not stained the whole mouse mammary gland possibly because of tissue penetration issues. The failure of some antibodies in the uDISCO procedure is possibly due to the fact that labelling by primary and secondary antibodies is performed immediately after fixation of the whole mammary gland, before reduction of the organ (and not in thin tissue section as in the classical immunohistochemistry procedure). This is a limit of the uDISCO method that we are now discussing in the revised manuscript.
Of note, laminin 5 is widely accepted as an essential component of the mature mammary basement membrane, and often used as a marker to assess basement membrane integrity. Like other basement membrane proteins, it is primarily produced by myoepithelial rather than luminal cells (see expression arrays, Kendrick et al 2008 BMC Genomics 9, 591; Lim et al 2009 Nat Med 15, 907-913). As shown in our previous work (Villeneuve et al, PNAS 2019), lentiviral infection predominantly targeted luminal cells in the organoids. In consequence, we do not most probably affect the production and the secretion of basement membrane by the myoepithelial cells.
Finally, in transplanted GFP$^+$ control organoids, we did not observe any breaching of the basement membrane (Figure 3 and Villeneuve et al, PNAS 2019), suggesting that lentiviral the infection and organoid transplantation procedures did not critically alter basement membrane deposition and organization.

2. *Efforts should be undertaken to show the individualized cells with higher resolution. Can they be classified as "Amoeboid", in line with previous work from the Condeelis group.*

We are grateful to the reviewer for this suggestion. However, we think it is too preliminary to make any strong conclusions regarding the cell migratory modes from our study. In addition, and as suggested by Referee #1, we have refocused our manuscript to meet the criteria of a technical resource, which is more centered towards the methodological aspects of the study, such as the ability to detect rare GFP$^+$or GFP-aPKCi$^+$ epithelial cells as well as the breaching of the basement membrane by GFP-aPKCi$^+$ epithelial cells. The detection and discrimination of amoeboid *versus* mesenchymal cells invading into the stroma during tumor progression remains a valid issue that is now discussed in the Discussion section of the revised manuscript.

3. *The frequency of individualized cells, their origin (terminal end buds, ducts etc.) and more detail on the time course of detachment should be provided. Time-gated controls are required to exclude that single cells are remnants from much earlier stages of ductal sprouting, where the basement membrane was incomplete, or caused by the wound healing response. This would rule out an artefact of the constitutive aPKCi overexpression combined with the surgery and gland reconstruction procedure.*

We agree with the reviewer on this point and initially we aimed to perform a longitudinal study to analyze the number of basement membrane-breaching GFP-aPKCi$^+$ cells. First, note that in Villeneuve et al, PNAS 2019 we provided images and quantification of GFP-aPKCi$^+$ cells in the epithelial monolayer or breaching the myoepithelial layer and the basement membrane. Of note, we identified some GFP-aPKCi$^+$ luminal cells still remaining in the epithelial cell layer with an intact basement membrane (Figure 3b - blue arrows). However, our longitudinal study attempt proved to be very challenging, probably due to the fact that once extruded from the mammary epithelium, rare GFP-aPKCi$^+$ cells may die due to the lack of survival factors. Therefore, at the moment it is difficult to address the frequency of the individual cells breaching the BM, as we do not know how many cells are infected, how many die… This point is also discussed below in Point 4 and is also added to the discussion. Long-term intravital imaging could provide an unambiguous answer to these questions; however, these experiments are out of the scope of this study that we refocused on methodological aspects. Also, noticeably, our analyses were performed between 5-9 weeks post-transplantation in order to have a fully regenerated mammary as shown in Figure 2B. The analysis of the GFP$^+$ cells showed an intact basement membrane confirming that the surgery and gland reconstruction procedure did not interfere with basement membrane formation and we never observed breaching of the basement membrane by GFP$^+$ cells. Also, the presence of GFP-aPKCi$^+$ epithelial cells is in itself a rare event—thus, this is not likely to interfere with global basement membrane deposition, in which ECM components are mainly secreted by myoepithelial cells (see point 1).

© 2020. Published by The Company of Biologists under the terms of the Creative Commons Attribution License (https://creativecommons.org/licenses/by/4.0/).

4. Lastly, the fate of these detached cells should be shown. It is within direct reach of this approach to show whether these cells survive and end up in distant organs, because the hematogenous route is expected to be intact. Technically, this analysis is critical to validate the suitability of the implantation model as spontaneous metastasis model. Biologically, the data would demonstrate, whether aPKCi signaling is sufficient to drive the whole metastatic cascade.

We appreciate the reviewer's suggestions and advices on how to strengthen the biological aspects of our study. Instead of elaborating in detail the biological consequences of aPKCi overexpression, we have refocused our manuscript on the technological aspects of this resource study with a large part of the discussion dedicated to technical advantages of our method in the context tumor progression or mammary gland development. However, we do not think that the overexpression of aPKCi will be enough to trigger the whole metastatic cascade; some other oncogenic mutations should most probably happen in order for cells to survive in the stroma. This issue is now discussed in the revised manuscript.

Minor points:

Fig. 2B should include zoomed images /details on intact duct and bud structure.

We provided a zoom in 2b at 5 weeks post transplantation. We analyzed the regenerated mammary gland from 5 to 9 weeks post transplantation. Also, it is worth mentioning that the presence of GFP^+ or $GFP\text{-}aPKCi^+$ epithelial cells remains a rare event based on the low m.o.i. infection procedure we have used and thus, it is not likely interfering with global formation of bud and duct structures. We do not see any major defect in regenerated mammary gland from GFP^+ or $GFP\text{-}aPKCi^+$ organoids.

Fig. 3B (e.g. zoom 2) and D (zoom 3) should also show orthogonal projections, to demonstrate that the individualized cells are fully detached from the strand. Single cell detachment is obvious from the sample rotations in the movies, but evidence should also be clear in the images.

We are very grateful to the reviewer for this suggestion and we provided some orthogonal projections in Figure 3b and 3d.

Second decision letter

MS ID#: JOCES/2019/242495

MS TITLE: A new pipeline for pathophysiological analysis of the mammary gland based on organoid transplantation and organ clearing

AUTHORS: Emilie Lagoutte, Clementine Villeneuve, Vincent Fraisier, Denis Krndija, Marie-Ange Deugnier, Philippe Chavrier, and Carine Rosse
ARTICLE TYPE: Tools and Resources

I am happy to tell you that your manuscript has been accepted for publication in Journal of Cell Science, pending standard ethics checks.

Reviewer 1

Advance summary and potential significance to field

The authors have satisfactorily addressed my previous comments.

Comments for the author

N/A.

© 2020. Published by The Company of Biologists under the terms of the Creative Commons Attribution License (https://creativecommons.org/licenses/by/4.0/).

Reviewer 2

Advance summary and potential significance to field

The revised manuscript tones down the biological claims, and expanded on the technical aspects of this work. It now provides a useful resource for mammmary explant culture, clearing and whole-sample imagung with subcellular resolution.

Comments for the author

None

© 2020. Published by The Company of Biologists under the terms of the Creative Commons Attribution License (https://creativecommons.org/licenses/by/4.0/).
